# Clinical Characteristics of Pregnancy-Associated Central Serous Chorioretinopathy in the Chinese Population

**DOI:** 10.1155/2021/5580075

**Published:** 2021-12-16

**Authors:** Jia Yu, Lei Li, Chunhui Jiang, Qing Chang, Gezhi Xu

**Affiliations:** ^1^Department of Ophthalmology, Eye & Ear Nose Throat Hospital of Fudan University, Shanghai 200031, China; ^2^Shanghai Key Laboratory of Visual Impairment and Restoration, Fudan University, Shanghai 200031, China; ^3^NHC Key Laboratory of Myopia (Fudan University), Shanghai 200031, China; ^4^Laboratory of Myopia, Chinese Academy of Medical Sciences, Shanghai 200031, China

## Abstract

**Purpose:**

To investigate the clinical characteristics of pregnancy-associated central serous chorioretinopathy (CSC) in the Chinese population.

**Methods:**

The medical records of patients diagnosed with pregnancy-associated CSC from February 2012 to October 2019 were retrospectively reviewed. Best-corrected visual acuity (BCVA), symptom duration, pregnancy-related medical information, and optical coherence tomography (OCT) images were collected.

**Results:**

Nine patients (11 eyes) were included. Five women were in their first pregnancy and four were in their second pregnancy, two of whom experienced CSC in their first pregnancy as well. The mean age was 35.00 ± 3.97 years. The mean symptom duration at the initial visit was 19.73 ± 13.65 days. The mean gestational age at the time of development of CSC was 27.11 ± 2.09 weeks. The mean BCVA (logarithm of the minimum angle of resolution (logMAR)) at the initial visit was 0.36 ± 0.18 (Snellen 20/45, range 20/100–20/25). All eyes showed subretinal hyperreflective fibrin on OCT images at the initial visit. Four patients (4 eyes) were lost to follow-up before fluid resolution. The mean BCVA at the final visit was logMAR 0.10 ± 0.15 (Snellen 20/25, range 20/50–20/20)). One eye in the oldest patient had persistent subretinal fluid at 26 months postpartum. The subretinal fluid resolved completely after half-dose photodynamic therapy (PDT); however, the ellipsoid zone at the fovea remained discontinuous at 30 months after half-dose PDT. The remaining six eyes all showed spontaneous resolution of subretinal fluid around delivery and regained intact ellipsoid zone.

**Conclusions:**

Pregnancy-associated CSC in Chinese developed mostly in the third trimester and usually recovered spontaneously around delivery with good final visual acuity. However, patients might require long-term follow-up until complete resolution of subretinal fluid and to detect recurrences. Half-dose PDT can be administered early if there is little reduction in the amount of subretinal fluid after delivery.

## 1. Introduction

Acute central serous chorioretinopathy (CSC), a common macular disease, usually presents with a well-circumscribed serous retinal detachment at the posterior pole on clinical examination and one or more leakage points at the level of the retinal pigment epithelium (RPE) on fluorescein angiography (FA) [1]. Pregnancy-associated CSC typically develops in the third trimester and is characterized by the onset of subretinal hyperreflective fibrin, spontaneous, and complete resolution of subretinal fluid around the time of delivery and good final visual acuity [2–16]. Cases have been reported in Caucasian, black, Native American, Hispanic, and Middle Eastern women [2–16]. Morikawa et al. reported on four pregnant Japanese women who developed CSC after onset of pre-eclampsia (PE) [17]. However, the clinical characteristics of pregnancy-associated CSC without PE in Eastern populations are not well described.

Historically, diagnosis of pregnancy-associated CSC was based on clinical presentation [2–6]. Rezai and Eliott demonstrated that optical coherence tomography (OCT) was useful for diagnostic and monitoring purposes in pregnant women with CSC without the risks to the fetus of exposure to fluorescein dye [7]. In this study, we used high-speed spectral-domain OCT to investigate the clinical characteristics of pregnancy-associated CSC in the Chinese population.

## 2. Materials and Methods

This retrospective study was approved by the Ethics Committee of the Eye and Ear, Nose and Throat Hospital, Fudan University, Shanghai, China, and adhered to the tenets of the Declaration of Helsinki. The medical records of patients who were diagnosed with pregnancy-associated CSC at Eye and Ear, Nose and Throat Hospital, Fudan University, between February 2012 and October 2019 were reviewed.

The clinical diagnosis of CSC was based on a finding of reduced visual acuity with or without metamorphopsia or micropsia and the presence of serous retinal detachment on fundus and OCT examinations. Data were collected for the affected eyes, including the symptom duration, best-corrected visual acuity (BCVA, measured using a standard Snellen chart and converted to logarithm of the minimum angle of resolution (logMAR) units for statistical analysis), intraocular pressure (using a noncontact tonometer), and findings on the slit-lamp biomicroscope. The number of the current pregnancy, any previous history of CSC, gestational age at the time of onset of the current episode of CSC, and blood pressure were also documented. The medical history of complication during pregnancy, systemic diseases, and intraocular diseases were also collected.

The exclusion criteria were as follows: hypertension, defined as systolic blood pressure >140 mmHg or diastolic blood pressure >90 mmHg; PE, defined as de novo hypertension present after 20 weeks of gestation combined with proteinuria or other maternal organ dysfunction [18]; renal disease; diabetes mellitus; any coexisting systemic disease; and any other intraocular disease, such as glaucoma, Posner–Schlossman syndrome, uveitis, white dot syndrome, or retinal vein occlusion.

All OCT images were obtained with a HD 5-line raster scan protocol (length 6 mm, spacing 0.075 mm, Cirrus HD-OCT, Carl Zeiss Meditec, Dublin, CA, USA) or with a line scan protocol (line scans of 30° composed of 100 averaged images; Heidelberg Spectralis OCT, Heidelberg Engineering, Heidelberg, Germany**)**. In each subject, this protocol was applied both vertically and horizontally and centered on the fovea of the affected eye(s). Using OCT, we analyzed subretinal hyperreflective fibrin and the number of changes in the RPE, including pigment epithelial detachment and RPE elevation in the active phase and the status of the ellipsoid zone (EZ) in the resolved phase.

The photodynamic therapy (PDT) protocol for CSC was performed with half the normal dose of verteporfin (Visudyne; Novartis AG, Bülach, Switzerland), that is, 3 mg/m^2^ verteporfin based on the rationale that a lower dose has less-severe collateral damage effects to the retina and choroid. Verteporfin was infused over 8 min, followed by delivery of laser at 689 nm at 10 min from the commencement of infusion to target the area of choroidal dilation and hyperpermeability. A total light energy of 50 J/cm^2^over 83 s was delivered to the angiographic leakage sites shown in FA and the area of choroidal hyperperfusion observed in indocyanine green angiography [19].

Descriptive statistics were calculated and are shown as means and frequencies. The Kolmogorov–Smirnov test was used to confirm the normality of the data. The data were analyzed using SPSS for Windows (version 21.0; IBM Corp., Armonk, NY, USA).

## 3. Results

**Figure 1:**
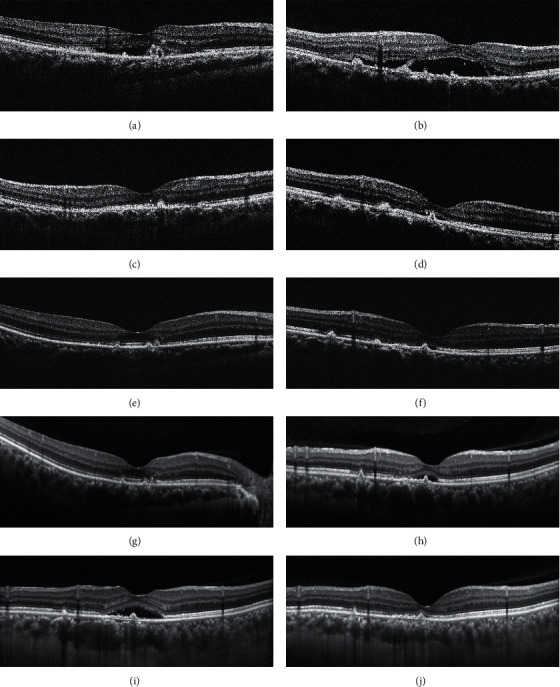
Optical coherence tomography images for case 3. (a), (c), (e), (g) Scans of the right eye. (b), (d), (f), (h), (i), (j) Scans of the left eye. (a, b) Images obtained at the initial visit (gestational week 31) showing subretinal fluid and subretinal hyperreflective fibrin at the sites of retinal pigment epithelium irregularity. (c, d) Images obtained 112 days later (49 days postpartum) showing complete resolution of subretinal fluid and a discontinuous ellipsoid zone in the right eye (c) and reduced but persistent subretinal fluid at the fovea in the left eye (d). (e, f) Images obtained 95 days later (144 days postpartum) showing an intact ellipsoid zone at the fovea in the right eye (e) and persistent subretinal fluid at the fovea in the left eye (f). (g, h) Images obtained 15 months later (20 months postpartum) showing an intact ellipsoid zone at the fovea in the right eye (g) and slightly increased subretinal fluid at the fovea in the left eye (h). (i) An image obtained 5 months later (25 months postpartum) showing significantly increased subretinal fluid at the fovea in the left eye. After a month's consideration, the patient finally agreed to administration of half-dose photodynamic therapy (26 months postpartum) and was then lost to follow-up for a further 30 months. (j) An image obtained 30 months after half-dose PDT (56 months postpartum) showing complete resolution of subretinal fluid at the fovea in the left eye but persistence of discontinuity of the ellipsoid zone at the fovea.

**Figure 2:**
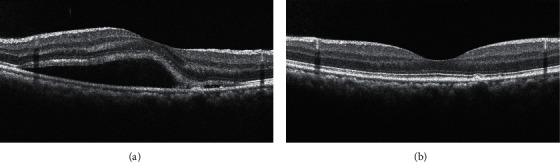
Optical coherence tomography images for case 7. (a) An image obtained at the initial visit (gestational week 31) showing subretinal fluid and subretinal hyperreflective fibrin at the site of retinal pigment epithelium irregularity. (b) An image obtained at the final visit (42 days postpartum) showing absolute resolution of subretinal fluid and an intact ellipsoid zone throughout the macula.

Nine patients (11 eyes) were included. Five women were in their first pregnancy and four were in their second pregnancy, two of whom experienced CSC in their first pregnancy as well. None had developed symptomatic CSC before their pregnancies. All patients had blood pressure within the normal range at the initial visit. No patient had any prior systemic disease. The mean patient age was 35.00 ± 3.97 years. The mean duration of symptoms at the initial visit was 19.73 ± 13.65 days. The mean gestational age at the time of development of CSC was 27.11 ± 2.09 weeks. The mean BCVA (logMAR) at the initial visit was 0.36 ± 0.18 (Snellen 20/45, range 20/100–20/25). All eyes showed subretinal hyperreflective fibrin on OCT images at the initial visit. Six of the 11 eyes (54.5%) showed multifocal RPE abnormal sites on OCT. They were all from the four elder patients with age of ≥35 y. Four patients (4 eyes) were lost to follow-up before fluid resolution. The mean BCVA (logMAR) at the final visit was 0.10 ± 0.15 (Snellen 20/25, range 20/50–20/20)). One eye, in the oldest patient in the series, had persistent subretinal fluid at 26 months postpartum that resolved completely after treatment with half-dose PDT; however, the ellipsoid zone at the fovea remained discontinuous at 30 months after half-dose PDT (Figure 1). Treatment was not performed sooner because the patient missed several follow-up visits. The remaining six eyes showed spontaneous resolution of subretinal fluid around the time of delivery and regained an intact EZ (Figure 2). The clinical data are given in Table 1.

## 4. Discussion

Consistent with previous studies, this study showed that pregnancy-associated CSC developed mostly in the third trimester and usually recovered spontaneously around the time of delivery with good final visual acuity [2–16].

Case-control studies have shown that pregnancy is an independent risk factor for development of CSC [20, 21]. Pregnancy dramatically affects the hypothalamic-pituitary-adrenal axis, leading to increased circulating cortisol levels during gestation [22, 23]. Plasma-free cortisol, salivary cortisol, and 24-hour urinary free cortisol were reported to reach peak levels during the third trimester and to return rapidly to baseline after delivery [22, 24]. The timing of development of CSC coincided with cortisol reaching peak levels, and the timing of resolution of CSC coincided with the decrease in cortisol levels. The overlap of the period between persistence of CSC and elevated cortisol levels support the hypothesis that pregnancy-associated CSC is related to a hypercortisolemic state via an endogenous mechanism [5]. However, the underlying mechanism remains unclear. Inhibition of collagen synthesis by cortisol may produce thinning of the capillary walls, resulting in increased capillary fragility and thus increasing the permeability of choroidal vessels [5, 25, 26].

Al-Mujaini and Wali reported the case of a woman who developed CSC in two consecutive pregnancies [12]. In the present study, four patients developed CSC in a second pregnancy, and two of these women had developed CSC during their first pregnancy. Although both women recovered spontaneously after the second pregnancy with an intact EZ and BCVA of 20/20, patients with pregnancy-associated CSC should be informed of the risk of recurrence in subsequent pregnancies.

Lopez-Yang and Garcia described a 40-year-old woman with pregnancy-associated CSC who had persistent subretinal fluid 20 months postpartum [27]. Similarly, in our study, one eye in a 42-year-old woman (the oldest in our series) had persistent subretinal fluid at 26 months postpartum. It has been suggested that overwhelming of the barrier function of the RPE contributes to the leak of fluid from the choroid to the subretinal space in CSC [28]. In aged human maculae, RPE cells were shown to increase in size and lose their regular hexagonal shape [29]. A study of aging monkeys observed elongation of the mitochondria in the RPE cells within the macular area that was an indicator of increased metabolic stress [30]. Moreover, RPE cells in the eyes of aged mice were found to decrease in number and undergo multinucleation because of failure of cytokinesis [31]. All these observations indicate that the repair capacity of the RPE may decrease with age, leading to persistent CSC [32]. Although half-dose PDT was shown to be effective in promoting resolution of subretinal fluid in this case, the EZ at the fovea remained discontinuous 30 months after half-dose PDT with little improvement in visual acuity. Therefore, although most eyes with pregnancy-associated CSC recover spontaneously around the time of delivery (Table 1) [2–13, 15, 16], all patients, especially those who are older, might require regular follow-up to confirm complete resolution of subretinal fluid. Half-dose PDT can be administered early if the amount of subretinal fluid shows little reduction after delivery.

Previous studies found subretinal hyperreflective fibrin to be common in the eyes with pregnancy-associated CSC, occurring in 50–100% of cases [2, 3, 5, 11], which is consistent with the finding in our study that all cases showed subretinal hyperreflective fibrin on OCT images at the initial visit. In a previous study, we reported that the eyes with subretinal hyperreflective fibrin in acute CSC had short durations of symptoms, with a mean of 18.85 days [33]. The mean duration of symptoms in the present study was comparable at 19.73 days. Therefore, the common finding of subretinal hyperreflective fibrin in the eyes with pregnancy-associated CSC could be related to a short duration of CSC symptoms rather than pregnancy itself.

Mothers who give birth at the age of 35 years or older are defined as being of advanced maternal age [34]. Coincidentally, the mean age of the patients in this study was 35.00 years. Moreover, the six eyes with multifocal RPE changes on OCT were all from the four patients elder than 35 years. With increasing age, the RPE cells in the macula of the normal human eye increase in size and lose their regular hexagonal shape [29]. It has also been suggested that the RPE in aged eyes may be vulnerable to increased hydrostatic pressure and choroidal hyperpermeability, leading to development of CSC [35]. Therefore, further research is required to clarify whether older age is a risk factor for pregnancy-associated CSC.

To the best of our knowledge, this is the first study to report on pregnancy-associated CSC without PE in Chinese women. However, this study has some limitations: (1) the sample size was small; (2) because some of the OCT images were not acquired in the enhanced depth imaging mode and the study was retrospective, the thickness of the choroid in the active and resolved phases was not investigated; (3) ultrawide field fundus autofluorescence and en face OCT were not acquired; thus, the RPE changes were not studied; (4) OCT angiography was not acquired; therefore, the macular neovascularization was not evaluated; (5) other physiologic and psychological risk factors associated with CSC, such as sleep apnea, obsessive compulsive, type A personality, and caffeine abuse, were not considered [20, 21, 36]. Prospective studies that include a larger sample size and multimodal imaging information would tell us more.

## 5. Conclusions

Pregnancy-associated CSC in Chinese developed mostly in the third trimester and usually recovered spontaneously at around the time of delivery with good final visual acuity. However, patients might require long-term follow-up until complete resolution of subretinal fluid and to detect recurrences. Half-dose PDT can be administered early if there is little reduction in the amount of subretinal fluid after delivery.

## Figures and Tables

**Table 1:** Demographic characteristics and clinical data of Chinese patients with pregnancy-associated central serous chorioretinopathy.

Case	Age (years)	BP at initial visit (mmHg)	Number of the current pregnancy	History of CSC in the first pregnancy	Eye	Gestational week at onset of current CSC (weeks)	Duration of symptoms of CSC at initial visit (days)	BCVA at initial visit (Snellen)	Number of RPE abnormal sites on OCT	Treatment	Follow-up duration	BCVA at final visit (Snellen)	Subretinal fluid at final visit	EZ status at fovea at final visit
1	34	120/79	1		OD	31 + 2	5	20/100	1	N/A	N/A	N/A	N/A	N/A
2	33	116/73	1		OS	26	14	20/25	1	No	PPD 70 days	20/20	None	Intact
3	42	105/70	1		OD	27	28	20/50	mf	No	PPD 144 days	20/20	None	Intact
					OS	27 + 2	26	20/50	mf	Half-dose PDT	PPD 56 months (after half-dose PDT 30 months)	20/50	None	Discontinuous
4	35	110/70	1		OD	29	30	20/60	mf	No	PPD 35 days	20/25	None	Intact
					OS	29	30	20/60	mf	No	PPD 35 days	20/30	None	Intact
5	36	100/80	2	Yes	OD	27 + 5	2	20/30	mf	No	GW 35	20/20	None	Intact
6	34	110/70	2	No	OS	28	3	20/40	1	N/A	N/A	N/A	N/A	N/A
7	29	121/71	2	Yes	OD	25 + 4	38	20/25	1	No	PPD 6 weeks	20/20	None	Intact
8	40	116/66	1		OD	24 + 2	7	20/50	mf	N/A	N/A	N/A	N/A	N/A
9	32	120/80	2	No	OD	27 + 4	34	20/50	1	N/A	N/A	N/A	N/A	N/A

BCVA, best-corrected visual acuity; BP, blood pressure; CSC, central serous chorioretinopathy; EZ, ellipsoid zone; GW, gestational week; mf, multifocal; N/A, not applicable; OCT, optical coherence tomography; PDT, photodynamic therapy; PPD, postpartum day; Y, yes.

## Data Availability

The medical records used to support the findings of this study are restricted by the Ethics Committee of Eye and Ear Nose Throat Hospital Fudan University in order to protect patient privacy. Data are available from the corresponding author for researchers who meet the criteria for access to confidential data.

## Abbreviations

BCVA: Best-corrected visual acuity
CSC: Central serous chorioretinopathy
EZ: Ellipsoid zone
FA: Fluorescein angiography
logMAR: The logarithm of the minimum angle of resolution
OCT: Optical coherence tomography
PDT: Photodynamic therapy
PE: Pre-eclampsia
RPE: Retinal pigment epithelium.

## References

[1] Klais C. M., Ober M. D., Ciardella A. P., Yannuzzi L. A., Schachat A. P. (2006). Central serous chorioretinopathy. *Retina*.

[2] Gass J. D. M. (1991). Central serous chorioretinopathy and white subretinal exudation during pregnancy. *Archives of Ophthalmology*.

[3] Sunness J. S., Haller J. A., Fine S. L. (1993). Central serous chorioretinopathy and pregnancy. *Archives of Ophthalmology*.

[4] Khairallah M., Nouira F., Gharsallah R., Chachia N. (1996). Central serous chorioretinopathy in a pregnant woman. *Journal Francais Ophtalmology*.

[5] Quillen D. A., Gass J. D. M., Brod R. D., Gardner T. W., Blankenship G. W., Gottlieb J. L. (1996). Central serous chorioretinopathy in women. *Ophthalmology*.

[6] Normalina M., Zainal M., Alias D. (1998). Central serous choroidopathy in pregnancy. *The Medical journal of Malaysia*.

[7] Rezai K. A., Eliott D. (2004). Optical coherence tomographic findings in pregnancy-associated central serous chorioretinopathy. *Graefe’s Archive for Clinical and Experimental Ophthalmology*.

[8] Mayo G. L., Tolentino M. J. (2005). Images in clinical medicine. Central serous chorioretinopathy in pregnancy. *New England Journal of Medicine*.

[9] Al-Mujaini A., Wali U., Ganesh A., Montana C. (2008). Natural course of central serous chorioretinopathy without subretinal exudates in normal pregnancy. *Canadian Journal of Ophthalmology*.

[10] Hirji N., Watt L., Richardson E. (2010). Central serous chorioretinopathy secondary to childbirth. *BMJ Case Reports*.

[11] Said-Ahmed K., Fawzy M., Moustafa G. (2012). Incidence and natural course of symptomatic central serous chorioretinopathy in pregnant women in a maternity hospital in Kuwait. *Middle East African Journal of Ophthalmology*.

[12] Al-Mujaini A., Wali U. (2014). Alternating central serous chorioretinopathy in two consecutive pregnancies. *Oman Journal of Ophthalmology*.

[13] Chakraborti C., Samanta S. K., Faiduddin K., Choudhury K. P., Kumar S., Mondal R. (2014). Bilateral central serous chorio-retinopathy in pregnancy presenting with severe visual loss. *Nepalese Journal of Ophthalmology*.

[14] Narayanan S., Anantharaman G., Gopalakrishnan M., Anthony E. (2015). Spectral domain optical coherence tomography-guided laser treatment of central serous chorioretinopathy in a pregnant woman. *RETINAL Cases & Brief Reports*.

[15] Maggio E., Polito A., Freno M. C., Pertile G. (2015). Multimodal imaging findings in a case of severe Central Serous Chorioretinopathy in an uncomplicated pregnancy. *BMC Ophthalmology*.

[16] Olusanya B., Oluleye T. (2015). Unilateral central serous chorioretinopathy in a pregnant Nigerian woman. *Nigerian Medical Journal*.

[17] Morikawa M., Cho K., Kojima T. (2017). Risk factors for central serous chorioretinopathy in pregnant Japanese women. *Journal of Obstetrics and Gynaecology Research*.

[18] Mol B. W. J., Roberts C. T., Thangaratinam S., Magee L. A., de Groot C. J. M., Hofmeyr G. J. (2016). Pre-eclampsia. *The Lancet*.

[19] Lai T. Y. Y., Chan W. M., Li H., Lai R. Y. K., Liu D. T. L., Lam D. S. C. (2006). Safety enhanced photodynamic therapy with half dose verteporfin for chronic central serous chorioretinopathy: a short term pilot study. *British Journal of Ophthalmology*.

[20] Chatziralli I., Kabanarou S. A., Parikakis E., Chatzirallis A., Xirou T., Mitropoulos P. (2017). Risk Factors for Central serous chorioretinopathy: multivariate approach in a case-control study. *Current Eye Research*.

[21] Ersoz M. G., Arf S., Hocaoglu M., Sayman Muslubas I., Karacorlu M. (2018). Patient characteristics and risk factors for central serous chorioretinopathy: an analysis of 811 patients. *British Journal of Ophthalmology*.

[22] Jung C., Ho J. T., Torpy D. J. (2011). A Longitudinal study of plasma and urinary cortisol in pregnancy and postpartum. *The Journal of Clinical Endocrinology & Metabolism*.

[23] Lindsay J. R., Nieman L. K. (2005). The hypothalamic-pituitary-adrenal axis in pregnancy: challenges in disease detection and treatment. *Endocrine Reviews*.

[24] Allolio B., Hoffmann J., Linton E. A., Winkelmann W., Kusche M., Schulte H. M. (1990). Diurnal salivary cortisol patterns during pregnancy and after delivery: relationship to plasma corticotrophin-releasing-hormone. *Clinical Endocrinology*.

[25] Genuth S. M., Berne R. M., Levy M. N. (1993). The adrenal glands. *Physiology*.

[26] Perkins S. L., Kim J. E., Pollack J. S., Merrill P. T. (2002). Clinical characteristics of central serous chorioretinopathy in women. *Ophthalmology*.

[27] Lopez-Yang C. E., Garcia C. A. (2015). Persistent unilateral central serous chorioretinopathy in a breastfeeding woman. *Case Reports*.

[28] Nicholson B., Noble J., Forooghian F., Meyerle C. (2013). Central serous chorioretinopathy: update on pathophysiology and treatment. *Survey of Ophthalmology*.

[29] Rashid A., Bhatia S. K., Mazzitello K. I. (2016). RPE cell and sheet properties in normal and diseased eyes. *Retinal Degenerative Diseases*.

[30] Gouras P., Ivert L., Neuringer M., Nagasaki T. (2016). Mitochondrial elongation in the macular RPE of aging monkeys, evidence of metabolic stress. *Graefe’s Archive for Clinical and Experimental Ophthalmology*.

[31] Chen M., Rajapakse D., Fraczek M., Luo C., Forrester J. V., Xu H. (2016). Retinal pigment epithelial cell multinucleation in the aging eye - a mechanism to repair damage and maintain homoeostasis. *Aging Cell*.

[32] Daruich A., Matet A., Marchionno L. (2017). Acute central serous chorioretinopathy. *Retina*.

[33] Yu J., Jiang C., Xu G. (2014). Study of subretinal exudation and consequent changes in acute central serous chorioretinopathy by optical coherence tomography. *American Journal of Ophthalmology*.

[34] Goisis A., Remes H., Barclay K., Martikainen P., Myrskylä M. (2017). Advanced maternal age and the risk of low birth weight and preterm delivery: a within-family analysis using finnish population registers. *American Journal of Epidemiology*.

[35] Yu J., Xu G., Chang Q. (2019). Risk factors for persistent or recurrent central serous chorioretinopathy. *Journal of Ophthalmology*.

[36] Mansour A. M., Koaik M., Lima L. H. (2017). Physiologic and psychologic risk factors in central serous chorioretinopathy. *Ophthalmology Retina*.

